# A Systematic Review to Summarize and Critically Appraise Existing Phenotype Libraries Using Electronic Health Records

**DOI:** 10.1002/pds.70378

**Published:** 2026-04-26

**Authors:** Sima Mohammadi, Cori Campbell, Miriam C. J. M. Sturkenboom, Tiago A. Vaz

**Affiliations:** ^1^ Department of Data Science & Biostatistics University Medical Center Utrecht (UMCU) Utrecht the Netherlands; ^2^ Department of Health Services Research and Policy The London School of Hygiene and Tropical Medicine London UK

**Keywords:** algorithm, electronic health records, library, phenotype

## Abstract

**Purpose:**

Pharmacoepidemiology and population health studies using electronic health care records (EHRs) must define study variables through available electronic data. These variables are operationalized through phenotypes, which are a defined set of criteria used to identify specific traits or medical conditions. There is diversity across phenotype libraries (collections of code lists or algorithms) which intend to standardize these sets of criteria. This review aimed to characterize the landscape of phenotype libraries and how phenotypes are constructed, validated, managed, and reused across research settings.

**Methods:**

We conducted a systematic review of existing phenotype libraries to appraise their attributes. We systematically searched three databases (Scopus, PubMed, and Web of Science) up to November 2025 to identify studies on key characteristics of phenotype libraries. The search combined Medical Subject Headings (MeSH) terms related to “electronic health record,” “phenotype algorithm,” and “phenotype library”. A structured hand search was performed to identify relevant web‐based resources without accompanying publications to ensure comprehensive inclusion of libraries available to date. We extracted information on library size, vocabularies, phenotype construction methods, validation practices, management, and portability.

**Results:**

Of 336 articles, 37 met eligibility criteria for full‐text review, of which 25 were excluded because they were not EHR‐based phenotype libraries (representing single algorithms, genomic resources, or study‐specific phenotypes rather than reusable libraries), leaving 10 unique libraries described across 12 articles. A structured hand search identified seven more libraries. In total, 17 phenotype libraries met the inclusion criteria, including Education and Child Health Insights from Linked Data (ECHILD) Phenotype Code List Repository, Centralized Interactive Phenomics Resource (CIPHER), Chronic Condition Data Warehouse (CCW), ClinicalCodes Library, Clinical Classifications Software Refined (CCSR), ComPLy, CALIBER (Health Data Research UK (HDR UK) Phenotype Library or CALIBER), Jigsaw Algorithm Repository (JAR), Manitoba Centre for Health Policy (MCHP) Concept Dictionary, Open CodeLists, Observational Health Data Sciences and Informatics (OHDSI) ATLAS, PheCode, Phenotype KnowledgeBase (PheKB), Phenotype Execution and Modeling Architecture (PhEMA) Workbench, PheMap, Sharing and Reusing Computable Phenotype Definitions (SharePhe), Value Set Authority Center (VSAC). Libraries varied substantially in scope, size, and phenotype representation, including rule‐based algorithms, probabilistic phenotypes, and standardized code groupings. Validation practices were heterogeneous and reported only for a subset of libraries. All the libraries utilized a web‐based platform and met at least the minimum requirements for library management, including phenotype definitions, metadata, and version control.

**Conclusions:**

We observed large variations in library construction and validation across diverse libraries built in varied EHR research settings. The transparency of phenotypes and creating computable phenotypes enhance portability and streamline the effective reuse of phenotypes for different systems.

## Key Points

- We identified 17 phenotype libraries. The libraries varied in phenotype construction, including rule‐based algorithms, probabilistic models, standardized code groupings, and aggregated platforms that harmonize phenotypes from multiple sources.
- The successful development of rule‐based and computable phenotypes relies on a combination of clinical expertise and programming skills. Some libraries support machine learning or NLP either for phenotype development (PheKB, CIPHER, PheMap, OHDSI ATLAS) or for metadata extraction and harmonization (ComPLy).
- Libraries such as CIPHER, OHDSI ATLAS, PhEMA, PheMap, JAR, and SharePhe support computable phenotypes, enhancing reusability across systems.
- Phenotype validation approaches varied widely, and only a minority of libraries reported formal performance metrics. All libraries supported basic portability through standardized vocabularies, metadata, and web‐based access.

## Plain Language Summary

Electronic health records (EHRs) contain real‐world information about patients' medical conditions, treatments, and test results. Researchers use this data to study diseases and improve patient outcomes. To this end, researchers must specify how to define specific conditions in EHR data. These definitions are called phenotypes. Phenotype libraries are platforms where such definitions are collected, documented, and shared, allowing researchers to reuse them and ensure consistency across studies. In this study, we reviewed existing phenotype libraries to understand how they are built and how they support health research. We found 17 libraries, each with unique features. Most use rule‐based methods to define conditions, and some use machine learning and natural language processing to construct phenotypes. All are accessible through web platforms and readable by both humans and computers, but not all include validation of their definitions. User interfaces vary across libraries. Our findings show that phenotype libraries play a key role in improving the reliability and reproducibility of research leveraging EHR data. We also suggest improvements to increase their accessibility, quality, and ability to work across different systems.

## 1 Introduction

The digitalization of individual‐level patient data has enabled large‐scale reuse of electronic health records (EHRs) for real‐world evidence (RWE) generation in pharmacoepidemiology for decades [1, 2]. EHRs capture myriad patient parameters, including medical history, diagnoses, prescriptions, procedures, and vaccination records [3] and provide insights into the efficacy, safety, and utility of medical interventions in medical research [2]. However, since these data are not collected for research purposes but are often primarily collected for healthcare delivery, billing, and public health monitoring, they may not be fit for the purpose of addressing research questions. To overcome data purpose and quality challenges, important advances have been made, such as standardization of data sources' metadata, data quality assessment tools, and increased transparency of protocols and reporting [4, 5, 6]. In traditional clinical trials, variables are specified pre‐hoc and subsequently observed for study participants via primary data collection to address the evidence gap in question. In modern real‐world data (RWD) investigations, study variables must be created using data already present in EHR systems, requiring identification and transformation steps, meaning that the transparency of data processing and analysis methods is crucial [4].

Rule‐based phenotypes are explicit logical criteria combining diagnosis codes, medications, procedures, laboratory thresholds, and temporal conditions using Boolean operators to identify patients with a given condition [7, 8]. Computable phenotypes are machine‐readable definitions designed for programmatic execution via explicit logical rules and structured criteria [9]. A phenotype library is a structured repository that stores phenotype definitions, metadata, logic, and implementation details to support transparency, reproducibility, and reuse across studies and institutions [5, 10]. Phenotype libraries support the need for transparency by incorporating metadata to help structure the various components of a phenotype, supporting the generation of robust evidence from RWD to guide clinical decision‐making and health policy [7]. The re‐use and sharing of phenotypes becomes particularly important when working with EHRs in multiple countries, which may operate in different healthcare systems, languages, and vocabularies. Therefore, phenotype libraries should have standardized documentation of phenotype algorithms based on a common clinical definition.

Phenotype libraries must be adaptable to changes in clinical practice and evolving knowledge standards [8]. Routine curation and updating are necessary to maintain the accuracy and relevance of phenotypes [10]. A phenotype library should be developed by combining clinical expertise and scientific programming to ensure both human and computer utility [7]. Library portability refers to the ability to transfer and apply phenotypes across different systems; designing phenotypes that are both computable and portable enhances their reusability [9]. This appraisal aims to describe the landscape of existing phenotype libraries and their features, including size, construction process, maintenance, management, and portability through data models.

## 2 Methods

### 2.1 Database Search Strategy

This systematic review followed the Preferred Reporting Items for Systematic Reviews (PRISMA) requirements [11]. We systematically searched Scopus, PubMed, and Web of Science up to November 2025 for studies describing the development or utilization of EHR‐based phenotype libraries. The literature search strategy was developed iteratively by two authors (TV, CC) through pilot searches and successive refinement of keyword combinations. During early iterations, broader terms such as “phenotype library” and “electronic health records” were tested without additional qualifiers and yielded a high proportion of irrelevant records, including genetic association studies, biobank resources, and single‐study phenotype definitions. To improve specificity, the final search strategy combined three core concept domains including: (i) “phenotype”, (ii) “library” or “repository” explicitly linked to “algorithm” development or phenotype “authoring”, and (iii) “electronic health data sources”, including electronic health and medical records, insurance claims, and real‐world data (Table 1). In addition to the structured database search, a complementary hand search was conducted to ensure completeness and capture potentially relevant records not retrieved through database search. This manual search involved screening reference lists of included studies, reviewing related project websites, and exploring relevant repositories in Google Scholar to identify non‐indexed evidence.

**TABLE 1:** Search terms used in a systematic search of Embase, MEDLINE, and Web of Science databases.

Database	Search terms	Number of articles identified
PubMed	(“phenotyp*”[All Fields]) AND	61
	(((“librar*”[All Fields] OR “repositor*”[All Fields]) AND “algorithm*”[All Fields])) OR “authoring”[All Fields]) AND	
	(“electronic health records”[MeSH Terms] OR	
	“electronic health record*”[MeSH Terms] OR	
	“electronic medical records”[MeSH Terms] OR	
	“electronic medical record*”[MeSH Terms] OR	
	“insurance claim*”[MeSH Terms] OR	
	“real world data”[MeSH Terms] OR	
	“RWD”[MeSH Terms] OR	
	(“electronic”[All Fields] AND “health”[All Fields] AND “records”[All Fields]) OR	
	(“electronic”[All Fields] AND “health”[All Fields] AND “record*”[All Fields]) OR	
	(“electronic”[All Fields] AND “medical”[All Fields] AND “records”[All Fields]) OR	
	(“electronic”[All Fields] AND “medical”[All Fields] AND “record*”[All Fields]) OR	
	(“claim*”[All Fields] AND “database”[All Fields]) OR	
	(“real”[All Fields] AND “world”[All Fields] AND “data*”[All Fields]) OR	
	“electronic health records”[All Fields] OR	
	“electronic health record*”[All Fields] OR	
	“electronic medical records”[All Fields] OR	
	“electronic medical record*”[All Fields] OR	
	“insurance claim*”[All Fields] OR	
	“real world data”[All Fields] OR	
	“RWD”[All Fields]	
Scopus	(TITLE‐ABS‐KEY (“phenotyp*”) AND TITLE‐ABS‐KEY (((“librar*” OR “repositor*”) AND “algorithm*”) OR “authoring”) AND TITLE‐ABS‐KEY (“electronic health records” OR “real world data” OR “rwd” OR “insurance claim” OR “electronic medical records” OR (“electronic” AND “health” AND “records”) OR (“electronic” AND “medical” AND “records”) OR (“electronic” AND “claim” AND “database”) OR “electronic health records”))	49
Web of Science	(ALL = (phenotyp*)) AND	226
	(((ALL = (librar*) OR ALL = (repositor*)) AND ALL = (algorithm*)) OR ALL = (authoring)) AND	
	(ALL = (electronic health records) OR (ALL = (claim) AND ALL = (database)) OR (ALL = (electronic) AND ALL = (medical) AND ALL = (records)) OR (ALL = (electronic) AND ALL = (health) AND ALL = (records)) OR ALL = (electronic health records) OR ALL = (electronic medical records) OR ALL = (real world data) OR ALL = (RWD) OR ALL = (insurance claim))	

*Note:* Updated searches run to November 2025.

Articles were screened independently by three authors (SM, TV, CC). Articles identified for full‐text review were assessed by two reviewers, and conflicts were resolved by consensus. Studies describing individual phenotypes, study‐specific phenotypes, genotype libraries, and biobanks were screened out as they were not within the scope of this review, which focused on reusable phenotype infrastructures that enable standardized storage and sharing across institutions and/or networks. Our inclusion criteria focused on articles that provided descriptions of the phenotype creation and curation processes, including the methodologies and tools used for phenotype development. Each library's online interface was consulted to confirm size (number of phenotypes) and extract the most recent key features. No language restrictions were applied to the database search results.

### 2.2 Data Extraction and Reporting

The summary characteristics of the included articles were extracted by three authors (SM, TV, CC). Extracted characteristics included library name and size; vocabularies; knowledge used to construct phenotypes (medical, programming, or other); whether the phenotype algorithms are readable by a human and/or computer; the phenotype validation process; library maintenance; portability; management; and the user interface.

## 3 Results

### 3.1 Study Characteristics

**FIGURE 1:**
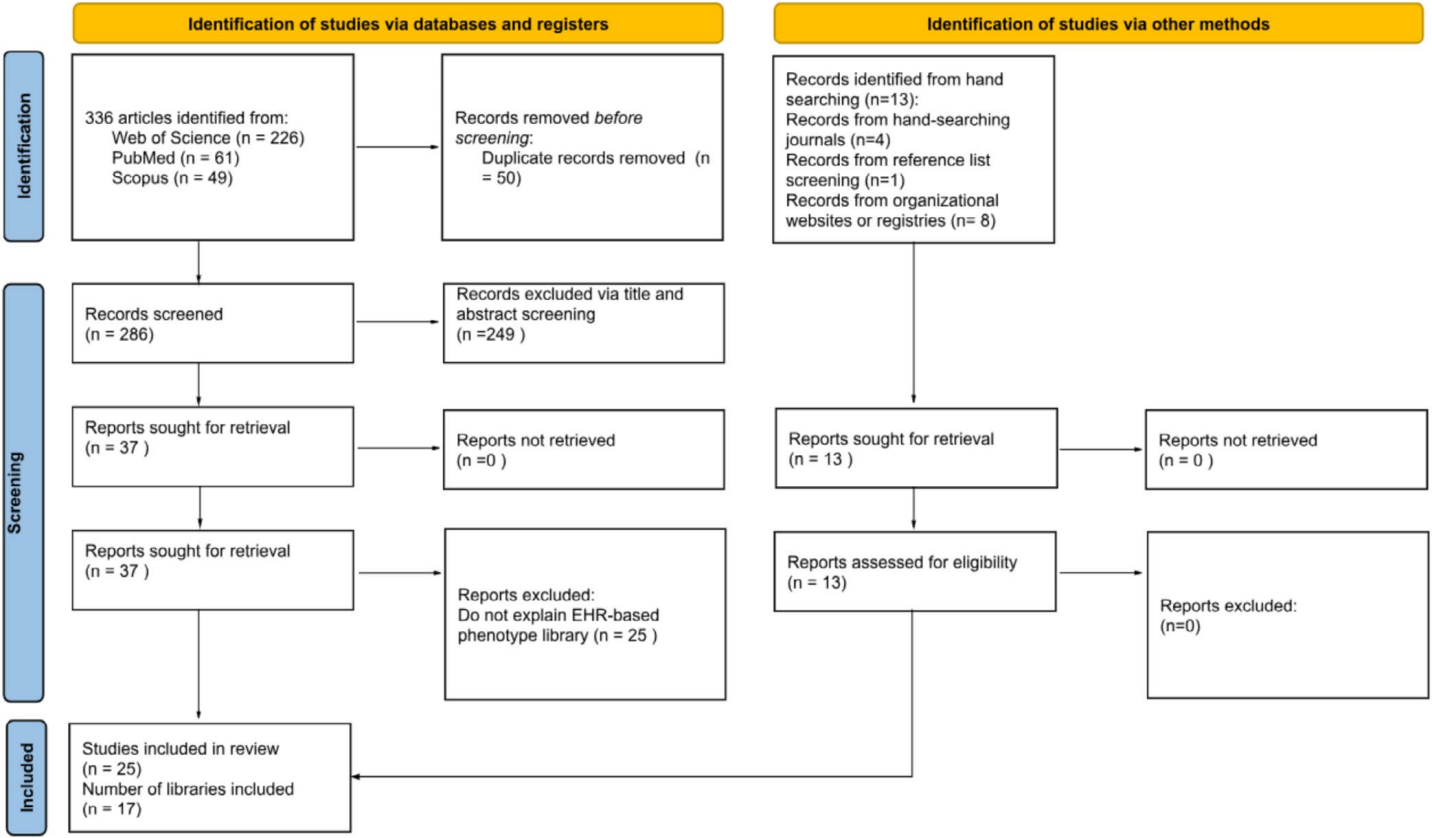
PRISMA Flow chart of selection of studies eligible for inclusion.

Our review identified a total of 336 articles, of which 286 remained after deduplication. Following title and abstract screening, 37 articles underwent full‐text reviews. Of these, 25 were excluded because they represented biobanks, genomic resources, or study‐specific phenotyping efforts rather than EHR‐based reusable phenotype libraries. A total of 12 articles were identified for final inclusion, including 10 unique phenotype libraries [12, 13, 14, 15, 16, 17, 18, 19, 20, 21, 22]. A hand search identified 13 additional resources belonging to seven unique libraries [23, 24, 25, 26, 27, 28, 29, 30, 31, 32, 33, 34, 35] (Figure 1).

In total, 17 individual phenotype libraries were identified, including Education and Child Health Insights from Linked Data (ECHILD) [21, 36], the Centralized Interactive Phenomics Resource (CIPHER) [13, 20, 37], Chronic Condition Data Warehouse (CCW) [22], the ClinicalCodes [19], Clinical Classifications Software Refined (CCSR) [24], ComPLy [23], the Health Data Research UK (HDR UK) Phenotype Library (also referred to as CALIBER for its data platform) [16], Jigsaw Algorithm Repository (JAR) [27, 28], the Manitoba Centre for Health Policy (MCHP) Concept Dictionary [14], the OpenCodeLists [15], the Observational Health Data Sciences and Informatics (OHDSI) ATLAS [17], PheCode [32, 33, 34], the Phenotype KnowledgeBase (PheKB) [12], the Phenotype Execution and Modelling Architecture (PhEMA) Workbench [18, 38], PheMap [31, 39], Sharing and Reusing Computable Phenotype (SharePhe) [25, 26], Value Set Authority Center (VSAC) [29, 30]. Libraries varied substantially in size, ranging from fewer than 100 phenotypes, as in ECHILD [36], CCW [22, 35], PheKB [40], PhEMA Workbench [21, 38], and SharePhe [25, 26], to thousands of phenotypes, as in CIPHER [13, 37] and CALIBER [16, 41], OHDSI [42], JAR [27, 28] (Table 2). Libraries supported a range of vocabularies, most commonly International Classification of Diseases (ICD‐10, ICD‐10‐CM, ICD‐9‐CM), SNOMED, procedural, and medication codes, largely drawn from the Unified Medical Language System (UMLS), with some also incorporating regional vocabularies [49], free‐text [13], and laboratory tests [14, 17, 18, 29]. Vocabularies used in each library are shown in Table S1.

**TABLE 2:** Summary characteristics of the existing phenotype libraries.

Category	Library	Computable phenotype support	Reusability/Execution context	Primary tools for phenotype creation	Library management & maintenance
Rule‐based phenotype libraries	OHDSI ATLAS [42, 43, 44]	Yes	Executable within OMOP CDM	ATLAS graphical interface (JSON cohort logic); optional ML via APHRODITE	Web‐based platform; versioning and sharing of cohort definitions, External collaboration
	PhEMA Workbench [18, 38]	Yes	Executable via CQL (FHIR‐based); portable across systems	CQL; reuse of PheKB phenotypes; VSAC value sets	GitHub‐based version control
	SharePhe [25, 26]	Yes	Executable across i2b2, OMOP, and PCORnet CDMs	CQL; i2b2 query framework; ACT ontology	Cloud‐based server; versioned phenotypes; prototype library
	JAR [27, 28]	Yes	Executable in OMOP CDM	ConceptQL (JSON); JAM/JAV/JOBS toolchain	Version tracking; open‐source; collaborative updating
	CIPHER [13, 37]	Yes	Executable via SQL; OMOP‐compatible	SQL‐based; ML‐assisted phenotype discovery	Web‐based platform with metadata, authorship, and versioning, Collaborative discussion
	CALIBER [16, 41]	Partial	Structured algorithms requiring local implementation	SQL‐based; open‐source R packages	Web portal; versioning; linkage to studies, Collaborative discussion
	PheKB [12, 40]	Partial	Human‐readable algorithms; selected computable workflows	Selected KNIME workflows; optional NLP integration	Drupal‐based platform; metadata, collaborative discussion, versioning
	CCW [22, 35]	Partial	Claims‐based algorithms requiring local execution	Predefined CMS rule‐based claims logic	Public documentation; centrally maintained by CMS
Probabilistic phenotypes	PheMap [31, 39]	Yes	Executable via Python; OMOP‐compatible	NLP‐based concept extraction; probabilistic scoring pipeline	Static knowledge base; reproducible pipeline (no user versioning)
Code‐based concept grouping repositories	VSAC [29, 30]	No	Downloadable value sets for reuse	Value set authoring and curation tools	Web interface; NLM‐maintained
	CCSR [24]	No	Code‐to‐category mappings	Centralized by AHRQ/HCUP	Periodic releases; centrally curated
	PheCode [32, 33, 34]	No	High‐throughput phenotyping in EHR/genomic studies	Automated ICD → pheCode mappings	Public repositories; versioned releases
	ECHILD [21, 36]	No	Downloadable codelists	—	Versioned CSVs; dual reviewer checks; community submissions
	ClinicalCodes [19, 45]	No	Downloadable and reusable codelists	SQL‐linked codelists; JSON metadata	Web portal; commenting; versioning
	OpenCodeLists [46, 47]	Yes	Executable within OpenSAFELY via ehrQL	ehrQL	GitHub‐based versioning; Dockerized workflows, Web‐based library
	MCHP Concept Dictionary [14, 48]	No	SAS‐based implementation within Manitoba data	SAS programming	Centrally maintained; version control
Harmonized aggregation platforms	ComPLy [23]	Yes	Machine‐readable, ontology‐based reuse	NLP‐assisted metadata extraction	FAIR‐compliant web portal; API access

Abbreviations: ACT = Accrual to Clinical Trials; AHRQ = Agency for Healthcare Research and Quality; APHRODITE = Automated PHenotype Routine for Observational Definition, Identification, Training, and Evaluation; API = Application Programming Interface; ATLAS = Automated Tool for Large‐scale Analytics Studies; CALIBER = HDR UK Phenotype Library; CCSR = Clinical Classifications Software Refined; CCW = Chronic Conditions Warehouse; CMS = Centers for Medicare & Medicaid Services; ComPLy = Computable Phenotype Library; CQL = Clinical Quality Language; ECHILD = Education and Child Health Insights from Linked Data; EHR = Electronic Health Record; ehrQL = Electronic Health Record Query Language; FAIR = Findable, Accessible, Interoperable, Reusable; FHIR = Fast Healthcare Interoperability Resources; HCUP = Healthcare Cost and Utilization Project; i2b2 = Informatics for Integrating Biology and the Bedside; ICD = International Classification of Diseases; JAR = Jigsaw Algorithm repository; KNIME = Konstanz Information Miner; MCHP = Manitoba Centre for Health Policy; ML = Machine Learning; NLM = National Library of Medicine; NLP = Natural Language Processing; OHDSI = Observational Health Data Sciences and Informatics; OMOP CDM = Observational Medical Outcomes Partnership Common Data Model; PCORnet = National Patient‐Centered Clinical Research Network; PheKB = Phenotype KnowledgeBase; PhEMA = Phenotype Execution and Modeling Architecture; PheMap = Phenotype Mapping framework; SAS = Statistical Analysis System; SQL = Structured Query Language; VSAC = Value Set Authority Center.

### 3.2 Phenotype Construction Process

Across the 17 libraries, we identified four main phenotype construction approaches, reflecting differences in purpose, data sources, and technical maturity: (1) rule‐based phenotypes, (2) probabilistic phenotypes, (3) code‐based concept grouping, and (4) aggregation platforms that harmonize phenotypes from multiple sources.

#### 3.2.1 Rule Based Phenotypes

Most libraries support rule‐based phenotype construction, where diagnostic codes, procedures, medications, laboratory thresholds, and temporal criteria are combined using Boolean logic. These libraries are CALIBER [41], CIPHER [37], OHDSI ATLAS [42], PheKB [40], PhEMA Workbench [38], SharePhe [25], JAR [27], and CCW [35], which provide transparent logical definitions, typically accompanied by metadata describing inclusion and exclusion criteria and details of algorithm construction. Phenotypes are presented in human‐readable formats and, in several cases, as computable representations executable within specific data models (Table 2). Several libraries support phenotype development using dedicated tools or programming languages. CALIBER translates phenotype algorithms into structured query language (SQL) queries and offers an open‐source R package for terminology management [16]. CIPHER [13] and OHDSI ATLAS [17, 42, 49] support machine learning (ML)‐based phenotype discovery. OHDSI employs the “Automated PHenotype Routine for Observational Definition, Identification, Training and Evaluation” (APHRODITE) R package to assist in training phenotype classifiers [17]. PhekB supports the use of natural language processing (NLP) for the identification of relevant features from free‐text EHR data. NLP is not implemented directly within the platform; however, researchers may integrate NLP into their workflows when using PheKB to extract EHR data from unstructured text [12]. Most PheKB phenotypes are human‐readable, with some computable representations, such as those developed with Konstanz Information Miner (KNIME), a tool which aids in developing computable phenotypes, enhancing reusability and portability [12]. The PhEMA Workbench uses clinical quality language (CQL) to encode inclusion and exclusion logic. PhEMA operationalizes some PheKB phenotypes, incorporates VSAC value sets as terminology components, translating them into executable CQL‐based definitions while also supporting native authoring of new computable phenotypes [38]. SharePhe is a prototype, CQL‐based computable phenotype library built on the i2b2 query framework and ACT ontology, offering syntactic/semantic validation, detailed metadata, and interoperability across common data models (e.g., OMOP, PCORnet) [25, 26]. JAR is a phenotype library derived from published studies, validated cohorts, quality‐measure programs, and code‐set groupings [28]. JAR operates through three integrated applications: the Jigsaw Algorithm Maker (JAM) for creating and editing algorithms, the Jigsaw Algorithm Viewer (JAV) for visualization, and the Jigsaw Observational Study Builder (JOBS) for assembling algorithms into complete observational study definitions. Algorithms can be selected from the repository or newly authored, and the study builder generates executable analysis datasets by compiling the associated queries [28]. CCW is a phenotype repository, providing algorithms that combine predefined diagnosis and procedure code lists with temporal and claims‐history criteria, such as required numbers of inpatient or outpatient encounters within specific look‐back periods, to identify chronic conditions in Medicare and Medicaid beneficiaries [22].

#### 3.2.2 Probabilistic Phenotypes

PheMap generates probabilistic phenotype definitions by integrating evidence from multiple biomedical knowledge sources, including UMLS concepts, the Mayo Clinic Patient Care and Health Information webpage, MedlinePlus, MedicineNet, and WikiDoc, using NLP and mapping methods rather than manual rule authoring. It links article‐derived concepts to ICD codes and ultimately to PheCodes, with associated probabilities that can be applied directly in OMOP‐formatted EHR data [31, 39]. PheMap leverages a broad range of real‐world data elements, including claims data, structured EHR data, laboratory results, medication, and free‐text clinical documentation, allowing the probabilistic model to capture diverse signals associated with each phenotype [31]. CIPHER and OHDSI also support ML‐based phenotype discovery; however, the phenotypes they store and share remain rule‐based and human‐defined, with ML methods used to assist phenotype extraction from EHR data. Therefore, both were classified as rule‐based phenotype libraries. However, PheMap phenotypes are defined through ML and NLP methods, without predefined, human‐authored rules; individuals are assigned probabilistic phenotype scores rather than deterministic case definitions.

#### 3.2.3 Code‐Based Concept Grouping

Standardized code or value‐set repositories included VSAC, CCSR, PheCode, ECHILD, ClinicalCodes, OpenCodeLists, and the MCHP Concept Dictionary. These resources provide curated groupings of clinically related codes rather than full rule‐based phenotype algorithms. VSAC is a publicly accessible repository of value sets maintained by the National Library of Medicine, providing diagnostic, procedure, laboratory, imaging codes, and electronic Clinical Quality Measures (eCQMs) [29, 30], but does not support full algorithmic phenotyping. Some value sets are provided through a partnership with the Mayo Clinic to support consistent terminology use [29, 30]. CCSR, developed by the Healthcare Cost and Utilisation Project (HCUP), is a standardized code‐grouping system that aggregates ICD‐10‐CM diagnosis codes and ICD‐10‐PCS procedure codes into clinically meaningful categories to support analyses of healthcare utilization, costs, and outcomes [24]. PheCodes were manually curated into clinically meaningful disease groupings, but the mapping of ICD‐9‐CM and ICD‐10‐CM codes to PheCodes was developed using a primarily automated pipeline based on General Equivalence Mappings (GEMs), Clinical Classifications Software (CCS), and UMLS crosswalks [34], and are widely used for high‐throughput phenotyping in large biobanks such as eMERGE and UK Biobank [32, 34]. ECHILD provides diagnostic and procedure codelists together with additional parameters (including birthweight and gestational age), derived from previously published sources which are curated and reformatted by the ECHILD team for consistency [21, 36]. The Open CodeLists Platform utilizes the electronic health records query language (ehrQL), a specialized language designed for querying data within the OpenSAFELY database, to enable reproducible cohort queries across heterogeneous UK EHR systems [15]. The MCHP library provides SAS‐based implementations of standardized concepts to support reuse in population‐level research [14].

#### 3.2.4 Harmonized Aggregated Phenotypes From Multiple Sources

ComPLy functions as a meta‐library rather than a primary phenotype authoring system. It aggregates and harmonizes phenotype definitions from multiple external repositories, including OHDSI, HDR UK, PheKB, PheMap, and PubMed, using NLP‐assisted metadata extraction and ontology‐based mapping to produce standardized, machine‐readable representations [23].

### 3.3 Phenotype Validation

All the libraries deployed a form of phenotype validation, although approaches varied across libraries (Table S2). Most libraries validate a subset of phenotypes rather than the full library. Phenotype validation is typically conducted by verifying individuals identified by specific phenotype algorithms against a clinical gold standard, often involving manual review of patient medical records, reporting performance metrics such as positive predictive value (PPV), sensitivity, and specificity. This approach was used by CALIBER [16], CIPHER [13], PheKB [12], PhEMA Workbench [18], PheMap [39] and PheCode [34]. Some libraries additionally employed genetic or external consistency validation, evaluating whether phenotypes replicated established genotype–phenotype associations or epidemiologic findings. This was implemented by CALIBER [16], PheMap [39], and PheCode [34] through genome‐wide association studies (GWAS) and phenome‐wide association studies (PheWAS). Beyond manual chart review and genetic validation, CALIBER applies a broader validation framework, including source concordance, case note review, consistency of risk factor‐disease associations on the basis of non‐EHR studies, consistency with findings from previous research, and comparisons with the external population [16]. PheMap further complements its validation strategy by comparing generated phenotypes with expert‐defined PheKB phenotypes [2]. PheKB limits access to phenotypes that are still under the development, testing, or validation phases, allowing visibility only to registered members of collaborative groups [7]. Some repositories focused primarily on terminology integrity and technical quality checks rather than clinical performance validation. VSAC performs iterative referential integrity checks to ensure correct mapping of codes to source vocabularies [30], while ECHILD applies clinical and technical verification of codelists without reporting formal validation metrics [36]. In the MCHP Concept Dictionary, selected concepts have undergone redefinition and validation, although validation methodologies are not systematically reported [14]. OHDSI uses APHRODITE to facilitate the creation and validation of phenotype definitions and has developed PheValuator as a framework to evaluate phenotype algorithm performance [17, 43]. Validation within OHDSI and CALIBER is decentralized, relying on independent execution of shared algorithms across distributed databases to assess reproducibility [16, 17]. Several libraries lack centralized clinical validation. JAR stores validation evidence when available but does not enforce a standardized validation framework [28]. SharePhe focuses on syntactic and semantic execution validation to ensure computable correctness [25, 26], and MCHP reports validation for selected concepts [14] (Table S2).

### 3.4 Library Portability, Management, and Maintenance

All libraries support at least minimal portability through downloadable codelists (Table 2). Most libraries are built and managed by their respective institutions; however, one exception is the ClinicalCodes library [19, 45], which has now migrated to the HDR UK library [16]. All libraries report version control mechanisms, including authorship, version identifier, and dating usage. Libraries are designed to facilitate phenotype evolution; however, they differ in the depth of phenotype management and metadata support. CIPHER offers structured phenotype definitions, codelists, rich metadata, authorship, creation dates, and version control, and supports sharing executable code or links to public repositories [20]. ClinicalCodes focuses primarily on transparent codelist sharing, offering citations for metadata, user comments, and automated phenotype downloads via an R package [19]. CALIBER supports storage of phenotype definitions, algorithms, metadata, and tools, with linkage to studies using the platform [41]. The MCHP library provides detailed operational definitions and programming codes to ensure methodological consistency [14]. OpenSAFELY supports maintaining codelists, sharing, version control, and community engagement through GitHub‐based workflows, ensuring reusability [15]. OHDSI ATLAS supports interactive cohort definition, with exportable JSON logic for reuse within OMOP‐based environments [18]. PheKB facilitates the cataloging, sharing, and discussion of phenotypes, hosting both descriptive documentation and executable logic, and uses the Drupal content management system to support metadata management, workflow optimization, and search functionality [12, 40]. ECHILD assigns unique identifiers and version numbers to each codelist, documents all modifications, and supports community submission of published codelists following dual independent review [21]. Sharephe provides a multi‐component interface for executing, browsing, and reusing computable phenotypes across institutions [25, 26]. JAR stores algorithms with versions and metadata, including validation evidence when available, and supports multiple algorithm classes (tested, published, and quality measures), with active modernization of its toolchain [27, 28]. Most libraries allow external users to register as contributors under defined contribution guidelines and provide advanced search functionality to support phenotype discovery [13, 19, 21, 28, 36, 37, 41, 42]. All libraries provide web‐based access, though user interfaces vary. Platform‐based libraries support interactive phenotype design and management, while code‐list repositories primarily support browsing and download. Libraries such as CIPHER [13], OHDSI ATLAS [17, 42], CALIBER [41], PhEMA Workbench [38], SharePhe [25], and JAR [27, 28] support interactive phenotype development and sharing, often with APIs or exportable and executable definitions. Some repositories, including VSAC [29, 30], CCSR [24], PheCode [32, 33], and ECHILD [36], primarily offer searchable interfaces for browsing and downloading standardized value sets or code lists. ComPLy provides a FAIR‐compliant (findable, accessible, interoperable, and reusable) ontology‐based portal for phenotype discovery and reuse [23]. CCW distributes phenotype definitions via public documentation and downloadable materials rather than an interactive platform [35] (Table 2).

## 4 Discussion

This review described 17 phenotype libraries supporting the construction and use of phenotypes for the secondary use of EHR. Libraries had diverse attributes and hosted phenotypes ranging from static code groupings to fully‐executable and portable phenotypes, reflecting differences in technical maturity, infrastructure, and research contexts [12, 13]. While all libraries met minimal portability requirements through standardized vocabularies, metadata, and human or machine‐readable formats, only a subset provided fully‐computable definitions for direct reuse across data models [8]. All identified libraries provide a structured platform that enables users to search, retrieve, and reuse phenotypes with associated metadata. Our findings align with prior studies highlighting that phenotypes may exist as codelists, rule‐based algorithms, NLP‐driven features, and/or trained classifiers [8].

High‐quality phenotypes are defined by their reproducibility, portability and validity [8]. Phenotype libraries should foster collaboration between authors and users, facilitating feedback and discussion [8]. Libraries such as ClinicalCodes, PheKB, CALIBER, CIPHER, ECHILD, and JAR support community interaction with transparency mechanisms, including discussion forums and metadata for citations. We have identified key areas for library innovation and improvement, including richer validation metadata, stronger community feedback mechanisms, support for NLP or ML‐based phenotypes, and wider availability of fully computable definitions [50]. A prior study showed that ML‐based phenotype classifiers developed using APHRODITE can be more efficient than rule‐based definitions [17], although sharing remains constrained by heterogeneity in EHR [17]. Therefore, the degree of computability and machine‐readability varies substantially across phenotype libraries and EHR data sources, reflecting differences in design maturity, technical infrastructure, and health systems.

Libraries such as OHDSI ATLAS, PheKB, JAR, and CIPHER provide fully‐executable phenotypes with structured logic and exportable representation, allowing direct implementation in compatible environments. Others publish detailed algorithm descriptions and codelists that are partially machine‐readable but may require local adaptation.

The scope and strengths of libraries reflect the healthcare systems and research contexts in which they were developed. CALIBER is aligned with UK‐linked health data and supports epidemiological and genomic research through extensively validated rule‐based phenotypes [16]. CIPHER, originating within the US Veterans Health Administration, provides richly annotated phenotypes tailored to large health‐system data and supports reuse in drug safety and genomic studies [20]. OHDSI ATLAS supports standardized cohort development across international networks using the OMOP Common Data Model [44], while PheKB emphasizes reproducibility in genomics and multi‐site studies [12]. VSAC, CCSR, and PheCode support consistent cohort construction in EHR‐based and genomic research [24, 30, 33], whereas CCW provides standardized claims‐based chronic condition surveillance [22, 35]. OpenCodelists [46], ClinicalCodes [19] and the MCHP [14] emphasize transparency, regional applicability, and methodological consistency. PhEMA Workbench and SharePhe demonstrate how CQL‐based approaches can enhance portability by quality measures and computable logics [18, 26, 38].

With regard to strengths and weaknesses of the various available libraries for RWE generation and pharmacoepidemiology research, various factors regarding the attributes of individual libraries must be considered. There are both library‐specific and wider health system factors which may impact the utility of a given library in addressing specific evidence gaps relating to pharmacological comparative effectiveness and post‐market safety. The health system settings in which specific libraries have been built may influence the suitability, specificity, and validity of phenotypes for robustly identifying medicine prescription and updates, receipt of specific medical procedures, and identification of outcomes and conditions of interest. Phenotype validity is imperative to regulatory evidence generation. Therefore, library‐specific validation procedures (including individual patient medical records review and linkage to centralized disease registries, along with other validation measures) warrants important consideration. The functionality to explore and compare phenotypes across diverse sources, compare validity metrics, and access transparent versioning and phenotype evolution information is also crucial. For these reasons, investigators should consider the clinical and geographic contexts of their research questions before choosing a library in which to explore relevant phenotypes.

We report a diverse range of library sizes. Variation in numbers of phenotypes available in libraries may arise from differences in repository scope and infrastructure maturity. Community‐driven libraries such as OHDSI and CIPHER host large numbers of phenotypes, as they support open submission models and broad coverage for large‐scale studies, leading to larger phenotype counts, whereas curated frameworks such as PheKB focus on a smaller set of phenotypes, as their primary scope is within genomics research contexts. Recognizing these contextual strengths may help researchers select phenotype resources aligned with their specific scientific objectives.

Phenotype validation varied widely across included libraries [16, 51]. Regulatory guidance recommends validation through patient chart review [51]. Our study highlights the diverse validation methods across libraries, ranging from manual chart reviews to cross‐EHR concordance [16], and automated approaches such as the PheValuator [17], with PheValuator demonstrating agreement with manual review. Chart review remains the gold standard for clinical accuracy, despite being labor‐intensive and potentially challenging to implement at scale [16, 17, 43]. Algorithm performance in PheKB can differ between sites, and validation is necessary to identify poorly performing algorithms [7]. Choice of validation method depends on the intended use of the EHR phenotype. For instance, algorithms used for disease surveillance prioritize high sensitivity, while those used in genetic association studies focus on achieving a high PPV to ensure that identified cases are true positives [16]. At present, many phenotypes lack formal validation evidenc, increasing the risk of misclassification and reducing reproducibility and quality of data across databases [52, 53]. Hybrid validation frameworks that combine manual review, cross‐dataset checks, and semi‐automated methods have the potential to improve transparency and reliability, particularly if accompanied by richer validation metadata and community feedback mechanisms [43, 50, 52]. Future work focusing on developing structured, multi‐level validation frameworks and quantifying their relative precision may serve to improve the quality of big data research [17, 52, 53, 54].

Despite the valuable content of existing libraries, there is currently no unified minimum standard for documentation. Next‐generation libraries may benefit from the adoption of standardized metadata and ontologies, machine‐readable representations, phenotype definitions, algorithm logic, validation evidence per data source, transparent versioning, and mechanisms for community review to improve interoperability and reuse [8, 12, 16, 49]. ComPLy illustrates this direction by implementing a FAIR‐compliant, ontology‐based infrastructure that aggregates phenotype definitions from multiple established repositories through NLP‐assisted metadata extraction and standardized, machine‐readable formats [23], demonstrating the feasibility of scalable, interoperable phenotype discovery and reuse.

This review was limited to phenotype libraries described in peer‐reviewed publications and targeted hand searches; consequently, some unpublished or non‐indexed resources may not have been included. Our review focused on describing library features and identifying gaps that provide direction for future work, but usability was not formally measured, and the ease with which phenotypes can be retrieved and deployed across new data environments could not be systematically compared. Future research is warranted to address this limitation by developing standardized frameworks for evaluating usability and implementation across heterogeneous data sources.

In conclusion, multiple phenotype libraries exist to date which contain diverse phenotypes which have been developed using diverse identification and validation methods, with interoperability, portability, and re‐usability differing across libraries. Future phenotype libraries should adopt standardized, FAIR‐aligned frameworks that ensure transparent documentation, computability, validation, and portability to support reproducible real‐world evidence generation.

## Author Contributions

C.C. and T.A.V. were responsible for the study conception and design. C.C., T.A.V., and S.M. were responsible for drafting the search terms, carrying out the systematic searches, screening and identifying the articles for inclusion and data extraction. S.M., C.C., T.A.V., and M.C.J.M.S. were responsible for manuscript drafting and review.

## Funding

Funding for this study was supported by a fellowship awarded by the Vaccine Monitoring Collaboration for Europe (VAC4EU) under award number 2023/0001 (Phenotype Representation Model: An International and Streamlined Approach to Enhance RWE Studies). VAC4EU is a European not‐for‐profit association that monitors vaccine coverage, safety, and effectiveness by leveraging real‐world data.

## Ethics Statement

The authors have nothing to report.

## Conflicts of Interest

The study funder approved the study design but did not play a role in the collection, analysis, or interpretation of the data. The funders were allowed to review and comment on the manuscript, but the authors retained full control of the final decisions, in compliance with the requirements of the ENCePP Code of Conduct [31]. Miriam C.J.M. Sturkenboom is the head of the Data Science and Biostatistics Department at University Medical Center Utrecht, which conducts studies for the European Medicines Agency and several vaccine manufacturers. All according to ENCEPP code of conduct.

## Supporting information


**Table S1:** Phenotype library Size and Vocabularies used in each library.
**Table S2:** Description of phenotype validation schemes for the included phenotype libraries.
