# Supplementary material for: A Systematic Review to Summarize and Critically Appraise Existing Phenotype Libraries Using Electronic Health Records
Source: Pharmacoepidemiol Drug Saf. 2026 Apr 26;35:e70378. doi: 10.1002/pds.70378 (PMC13110926; doi:10.1002/pds.70378)
Supplement: Supplementary file 1 — Table S1: Phenotype library Size and Vocabularies used in each library. Table S2: Description of phenotype validation schemes for the included phenotype libraries. [file PDS-35-e70378-s001.docx]

**Supplementary Table 1. Phenotype library Size and Vocabularies used in each library**

| **Library** | **Vocabulary** | **Library Size*** | **Library Link** |
| --- | --- | --- | --- |
| ECHILD | ICD-10 and OPCS-4 | 13 code lists | <https://code.echild.ac.uk/> |
| CIPHER | ICD-10-CM, ICD-10- PCS, CPT, Clinical stop, code, free-text, LONIC, NDC | ~6,857 phenotypes | <https://phenomics.va.ornl.gov/web/cipher/search> |
| CCW | ICD-9-CM, ICD-10-CM, CPT, HCPCS | ~60–65 conditions | <https://www.cms.gov/data-research/research/statistical-resources-dually-eligible-beneficiaries/data-tools-researchers> |
| ClinicalCodes | READ, OXMIS, ICD-9, SNOMED, CPRD, ICD-10, product/medical code, BNF code, OXMIS | 134310 clinical codes deposited ~ 670 code lists | <https://clinicalcodes.rss.mhs.man.ac.uk/> |
| CCSR | ICD-10-CM, ICD-10-PCS, CPT, HCPCS, ICD-9-CM | >530 diagnosis categories; >320 procedure categories | <https://hcup-us.ahrq.gov/toolssoftware/ccsr/ccs_refined.jsp> |
| ComPly | 46 coding system including (ICD9,  ICD9-CM, Read V2, ICD10, MED codes, SNOMED-CT) | ~3,538 phenotypes | <https://phenotypelibrary.org/> |
| CALIBER | ICD-9-CM, ICD-10, ICD-11, READ, CTV3, OPCS, SNOMED CT, BNF, GPRD product, OXMIS, Multilex, CTV3, ICPC2 and 4 other coding systems | 291,078 clinical codes  1,296 phenotypes; 2,353 code lists | <https://phenotypes.healthdatagateway.org/> |
| JAR | ICD-9, ICD-9-CM, ICD-10, ICD-10-CM, CPT, HCPCS, Read codes | ~1,180 algorithms | <https://public.jigsaw.io/> |
| MCHP | ICD10-CA, CCI, ICD-9-CM, Tariff, procedure, Medication, laboratory records | >373 concepts | <http://mchp-appserv.cpe.umanitoba.ca/search.php> |
| Open CodeLists | SNOMED CT, ICD-10, READ, CTV3, BNF | >1,700 code lists | <https://www.opencodelists.org/> |
| OHDSI ATLAS | SNOMED, ICD-10-CN, ICD-10-CM, ICD-9-CM, ICD-10, LONIC, and 45 other vocabularies | User-defined cohorts and concept sets (no fixed size) | <https://atlas-demo.ohdsi.org/#/home> |
| PheCode | ICD-9-CM and ICD-10-CM | 1,867 phecodes | <https://phenotyping.org/home> |
| PheKB | ICD-9, CPT,  lab, medication codes | ~88 phenotypes | <https://phekb.org/> |
| PhEMA Workbench | RXNORM, CPT, ICD-9-CM, ICD-9, LONIC | ~32 phenotypes | <https://github.com/PheMA/phema-workbench-app> |
| PheMap | ICD9-CM, ICD10-CM , SNOMED CT, CPT, LOINC, and RxNorm | ~1,400 phenotypes | <https://www.vumc.org/cpm/phemap> |
| Sharephe | SNOMED CT, ICD-9, ICD-10, LOINC, RxNorm, CPT | 35 phenotypes | <https://sharephe.dbmi.pitt.edu/> |
| VSAC | CDCREC, CPT, CVX, HCPCS, Health CareProvider Taxonomy, HSLOC, ICD10CM, LOINC, NCI, RXNORM, SNOMEDCT, SOP, UCUM, HL7 Code System, Act Code, Act Mood, Act Priority, Act Reason, Act Relationship Type, Act Status, Address Use, Confidentiality, Data Absent Reason, Entity Name Part Qualifier, Language Ability Mode, Language Ability Proficiency, Marital Status, MediaType, Null Flavor, Observation Interpretatin, Observation Value, Participation Function, Participation Type, Religious Affiliation, Role Class, Role Code | ~17,088 value sets | <https://vsac.nlm.nih.gov/> |

*Sizes are approximate and reflect publicly reported counts at the time of review (November 2025). A code list refers to a curated set of standardized clinical codes (e.g., ICD, SNOMED CT, CPT) representing a specific clinical concept or component of a phenotype. A phenotype may consist of more than a single code list. In many libraries, phenotypes are defined as algorithms that combine multiple code lists with explicit logical rules, temporal criteria, and other inclusion or exclusion conditions.

BNF: British National Formulary; CCI: Canadian Classification of Health Interventions; CDCREC: CDC Race and Ethnicity Code Set; CPT: Current Procedural Terminology; CPRD: Clinical Practice Research Datalink; CTV3: Clinical Terms Version 3; CVX: Vaccine Administered Code Set; Free-text: Unstructured clinical text; GPRD: General Practice Research Database; HCPCS: Healthcare Common Procedure Coding System; HL7: Health Level Seven International; HSLOC: Healthcare Service Location Code; ICD-9: International Classification of Diseases, Ninth Revision; ICD-9-CM: International Classification of Diseases, Ninth Revision, Clinical Modification; ICD-10: International Classification of Diseases, Tenth Revision; ICD-10-CA: International Classification of Diseases, Tenth Revision, Canada; ICD-10-CM: International Classification of Diseases, Tenth Revision, Clinical Modification; ICD-10-CN: International Classification of Diseases, Tenth Revision, China; ICD-10-PCS: International Classification of Diseases, Tenth Revision, Procedure Coding System; ICD-11: International Classification of Diseases, Eleventh Revision; ICPC-2: International Classification of Primary Care, Second Edition; LOINC: Logical Observation Identifiers Names and Codes; MED codes: CPRD medical codes; Multilex: Multilex Drug Dictionary; NCI: National Cancer Institute Thesaurus; NDC: National Drug Code; OPCS: Office of Population Censuses and Surveys Classification of Surgical Operations and Procedures; OXMIS: Oxford Medical Information System; Procedure codes: Procedural classification systems; READ: Read Clinical Classification System; RxNorm: Normalized drug nomenclature; SNOMED CT: Systematized Nomenclature of Medicine – Clinical Terms; SOP: Standard Operating Procedure; Tariff codes: Billing or reimbursement tariff classifications; UCUM: Unified Code for Units of Measure.

CALIBER:  HDR UK Phenotype Library; CCW: Chronic Conditions Data Warehouse; CCSR: Clinical Classifications Software Refined; CIPHER: Centralized Interactive Phenomics Resource; ClinicalCodes: ClinicalCodes Repository (University of Manchester); ComPly: Computable Phenotype Library; ECHILD: Education and Child Health Insights from Linked Data; JAR: Jigsaw Algorithm repository; MCHP: Manitoba Centre for Health Policy; OHDSI ATLAS: Observational Health Data Sciences and Informatics – ATLAS; Open CodeLists: Open and Shareable Clinical Code Lists; PheKB: Phenotype KnowledgeBase; PhEMA Workbench: Phenotype Execution and Modeling Architecture Workbench; Sharephe: Sharing and Reusing Computable Phenotype; VSAC: Value Set Authority Center.

**Supplementary Table 2. Description of phenotype validation schemes for the included phenotype libraries**

| **Library** | **Validation methods** |
| --- | --- |
| **CIPHER** | Clinical validation using manual chart review as gold standard; comparison against patient-reported data; external consistency testing through replication of known associations (e.g. GWAS findings) |
| **CALIBER (HDR UK Phenotype Library)** | Multi-layered validation framework including: (1) cross-EHR source concordance, (2) case-note review, (3) consistency of risk factor–disease associations with non-EHR studies, (4) consistency with prior prognosis research (e.g. survival curves), (5) comparison across external populations |
| **PheKB** | Clinical validation of selected phenotypes via manual chart review across eMERGE sites; technical validation using a Ruby-based service to identify structural and content errors in phenotype implementations |
| **OHDSI ATLAS** | Gold-standard comparison for selected phenotypes; semi-automated validation using PheValuator, which estimates phenotype performance using a probabilistic synthetic gold standard |
| **PhEMA Workbench** | Structured validation protocol with SQL-based random sampling of cases and non-cases followed by manual chart review against clinical gold standards |
| **PheMap** | Validation through comparison with expert-defined PheKB phenotypes; selected phenotypes additionally validated by manual chart review and replication of known genetic associations using GWAS/PheWAS |
| **PheCode** | Clinical validation via manual chart review of case/control assignments; genetic validation through replication of known SNP–phenotype associations from GWAS catalogs |
| **ECHILD Code List Repository** | Clinical review and technical verification of curated code lists; focus on correctness and applicability rather than performance estimation |
| **VSAC** | Terminology-level referential integrity checks to identify invalid, obsolete, or mismapped codes; no clinical validation |
| **JAR (Jigsaw Algorithm Repository)** | No centralized validation framework; validation evidence stored for individual algorithms when provided by original authors |
| **SharePhe** | Syntactic and semantic execution validation to ensure computable correctness of phenotype logic across systems |
| **MCHP Concept Dictionary** | Validation reported for selected concepts; detailed methods not systematically documented |
| **ClinicalCodes** | No formal centralized validation; relies on citation of published sources and community transparency |
| **Open CodeLists** | No clinical validation; technical reproducibility ensured through versioning, GitHub workflows, and execution context |

CIPHER, Centralized Interactive Phenomics Resource; CALIBER, ClinicAl research using LInked Bespoke studies and Electronic health Records (HDR UK Phenotype Library); EHR, electronic health record; eMERGE, Electronic Medical Records and Genomics Network; GWAS, genome-wide association study; HDR UK, Health Data Research UK; JAR, Jigsaw Algorithm Repository; MCHP, Manitoba Centre for Health Policy; OHDSI, Observational Health Data Sciences and Informatics; PheKB, Phenotype KnowledgeBase; PhEMA, Phenotype Execution and Modeling Architecture; PheWAS, phenome-wide association study; SNP, single-nucleotide polymorphism; SQL, Structured Query Language; VSAC, Value Set Authority Center.
